# The Benefits of Cooperative Inquiry in Health Services Research: Lessons from an Australian Aboriginal and Torres Strait Islander Health Study

**DOI:** 10.1177/27551938231221757

**Published:** 2023-12-25

**Authors:** Toby Freeman, Tamara Mackean, Juanita Sherwood, Anna Ziersch, Kim O’Donnell, Judith Dwyer, Deborah Askew, Madison Shakespeare, Shane D’Angelo, Matthew Fisher, Annette Browne, Sonya Egert, Vahab Baghbanian, Fran Baum

**Affiliations:** 1 Stretton Health Equity, 1066 The University of Adelaide, Adelaide, Australia; 2 College of Medicine and Public Health, 1065 Flinders University, Adelaide, Australia; 3 1994 University of Technology Sydney, Ultimo, Australia; 4 The University of Queensland, Brisbane, Australia; 5 The University of British Columbia Faculty of Applied Science, Vancouver, Canada; 6 Southern Qld Centre of Excellence in Aboriginal and Torres Strait Islander Primary Health Care - Inala Indigenous Health, Queensland Health, Inala, Australia; 7 Central Australian Aboriginal Congress, Alice Springs, Australia

**Keywords:** Indigenous health, health services research, qualitative research

## Abstract

Health services research is underpinned by partnerships between researchers and health services. Partnership-based research is increasingly needed to deal with the uncertainty of global pandemics, climate change induced severe weather events, and other disruptions. To date there is very little data on what has happened to health services research during the COVID-19 pandemic. This paper describes the establishment of an Australian multistate Decolonising Practice research project and charts its adaptation in the face of disruptions. The project used cooperative inquiry method, where partner health services contribute as coresearchers. When the COVID-19 pandemic hit, data collection needed to be immediately paused, and when restrictions started to lift, all research plans had to be renegotiated with services. Adapting the research surfaced health service, university, and staffing considerations. Our experience suggests that cooperative inquiry was invaluable in successfully navigating this uncertainty and negotiating the continuance of the research. Flexible, participatory methods such as cooperative inquiry will continue to be vital for successful health services research predicated on partnerships between researchers and health services into the future. They are also crucial for understanding local context and health services priorities and ways of working, and for decolonising Indigenous health research.

There is a growing emphasis in health services research on treating health services as complex adaptive organizations embedded in a local context, and subsumed within a wider, complex health system and political context.^1–3^ To complement research such as intervention trials to improve aspects of clinical care, there is a recognition that health services need to be studied within their local context to understand how they operate and how they balance policy and funding directives and regulations, organizational values, and local community needs.^2,4–6^ This need for the research to take account of the local context is particularly critical for Indigenous health services in Australia and other former colonies as these services tend to have a strong focus on responding to the unique needs of local communities and seeking to provide culturally safe practices to facilitate Indigenous communities’ meaningful access to holistic health and well-being services. This focus on serving the local community in a culturally safe manner requires tailoring all aspects of organizational activity^7–9^ towards a two-way model,^10^ prioritizing Indigenous knowledge. In this paper, we use Indigenous peoples when referring to global Indigenous populations, Aboriginal and Torres Strait Islander peoples when referring to Australia specifically, and Aboriginal when it matches how particular health services self-identify (such as the Aboriginal community-controlled health sector.

Understanding this local context and services’ ways of working requires in-depth research methods that must rest on close partnerships with the health services that are the topic of the research.^2^ However, the health system, including primary health care (PHC), is responding to constant changes through political shifts in priorities and funding gaps, often has very constrained resources, and is driven by goals that may not always align with research participation.^7^ For Indigenous health service research, culturally led collaborative relationships between researchers and health services is vital to generate meaningful research outcomes, and to facilitate trust.^11,12^ This trust must be grown through researchers’ recognition of the wide-reaching detriment Indigenous peoples continue to experience as a result of ongoing legacies of colonization, and the nurturing of respectful endeavors to further advance the successful work that is achieved in and by Indigenous health services, their workers, and respective communities. This trust needs to be fostered in the face of a track record of often exploitative research conducted on Indigenous peoples that frequently brought little benefit to Indigenous peoples.^13,14^ Growing and sustaining trustful collaborative relationships requires a great deal of time beyond what is estimated or covered by the grant funding, and the commitment of researchers to spend the time needed to grow meaningful relationships, not simply token research connections. The demanding pace of work required in both the research and health services world can undermine the capacity to be responsive to local needs, timelines, and priorities.^5^

Working in partnership with health services is particularly important given the disruption and challenges the COVID-19 pandemic has brought. In a survey by Research Australia^15^ of 304 Australian health and medical researchers, 80 percent reported COVID-19 adversely affected their research, including interruptions to participant recruitment, not being able to access health service research sites, and not being able to travel interstate or internationally for research. COVID-19 is especially a concern for Aboriginal and Torres Strait Islander health services research, as the social and political determinants of Indigenous health mean that Indigenous peoples globally have been at higher risk of contracting COVID-19, having severe symptoms, and dying from COVID-19.^16^ In Australia, Aboriginal and Torres Strait Islander health services have played a pivotal and leading role in protection of the health and well-being of Aboriginal and Torres Strait Islander peoples during the pandemic.^17,18^

Internationally, a small number of studies have discussed similar deleterious impacts of COVID-19 on research^19^ and cited challenges including clinical researchers being redeployed to COVID-19 duties, health service staff being overburdened and burned out to the point where they lacked the capacity to participate in research, the need to adapt to virtual data collection methods, and ethics committees deprioritizing research that is not directly relevant to the COVID-19 pandemic.^20–23^ These factors have presented challenges for researchers involved in non-COVID-19-related studies, as they contend with delays in approvals, reduced resources, and a reevaluation of their research objectives and methodologies. There has been a particular focus on the negative repercussions noted for early career researchers, who may be in precarious employment, are seeking to learn broad research skills, benefit most from networking, and need to produce outputs to secure ongoing employment.^20,21,23,24^

There are few studies of health services research adapting to COVID-19. Palese, Papastavrou, and Sermeus^20^ describe decision-making regarding how much to alter the original research design, and how to do so while protecting rigor, dealing with “incoherence” and “frailty” in the project, and the impact of the pandemic on researchers, such as needing to care for family, dealing with the stress and uncertainty, and working from home. Spagnolo, Gautier, Seppey, and D'Souza^21^ describe the impact on several health service research projects, including how they adapted to virtual data collection methods, and in one case, a student who considered completely changing to a more feasible PhD topic.

Beyond COVID-19, health services research adapted and will continue to need to adapt to other disruptions. Climate change-induced severe weather events such as fires, cyclones, and floods (we avoid the term natural disasters^25^) are becoming more frequent.^26^ These severe weather events directly result from colonial tenure and contribute to First Nations poorer health outcomes.^27^ Australia has seen severe bushfires and extreme flooding in recent years, and there are warnings of more frequent compound hazards—either concurrent or consecutive multiple severe weather events.^28^ These severe weather events place a heavy burden on local health systems and their respective communities, especially when combined with the COVID-19 pandemic,^29,30^ and are likely to interrupt and further complicate health services research.

This paper describes the establishment of an Australian multi-state research project, the Decolonising Practice project, and charts its adaptation in the face of the COVID-19 pandemic and severe weather events and the lessons derived from these experiences. The project entails a collaborative partnership between researchers and five Aboriginal and Torres Strait Islander primary health care (PHC) services examining how these services practice in decolonising ways. Decolonising practice refers to ways of working that aim to unpack the layers of imposed political determinants that have resulted from colonial tenures and seek to mitigate the adverse health effects stemming from ongoing colonization. This involves confronting power imbalances, addressing systemic racism, and adopting a strengths-based approach.^31,32^ It requires a “bureaucratic, cultural, linguistic and psychological divesting of colonial power”.^33^^, p. 98^ To understand better what decolonising practice looks like on the ground, we sought to study PHC services’ governance, staffing and interactions with their communities, and how they deliver treatment, disease prevention, and health promotion activities. We discuss how the collaborative partnership with health services subsequently supported the team's ability to respond and adapt the research to the COVID-19 pandemic and other disruptions.

The intent of this paper is to present the strengths and challenges of undertaking this large and complex health services research project in Australia during the COVID-19 pandemic, and during a series of severe weather events, to illustrate the benefits of collaborative approaches to health services research in times of significant change and difficulties.

This paper focuses on one element of this broader project—the value-add of designing a collaborative inquiry approach to health systems research. In Part One we outline the initiation of the project, how we partnered with services, and we describe the research design. In Part Two we consider how the research design and governance helped us navigate COVID-19 and other concerns and disruptions as the research progressed. We intend for this account to inform future research groups seeking to establish partnership-based research projects with health services, in both Indigenous and non-Indigenous health service contexts.

## Part One: Establishment of the Research Project

The Decolonising Practice project was based on the growing literature in Australia on decolonising practice in Aboriginal and Torres Strait Islander health care, particularly the work of Professor Juanita Sherwood.^34–37^ The project was part of a long-standing research program, building on a five-year project led by Professor Fran Baum (see for example^9,38^) that also partnered with seven PHC services (five non-Indigenous, and two Aboriginal PHC services).

Given our research topic of decolonising practice, it was essential to reflect that aim through decolonising research methods that focused on listening, growing trust, developing partnerships; emphasizing strengths-based approaches to counter the prevailing deficit discourse of Aboriginal and Torres Strait Islander health^39^; listening to and privileging Aboriginal and Torres Strait Islander voices; and acknowledging and exploring the power imbalances that exist in health systems and in research. Ethical Indigenous health research needs to be responsive to Indigenous cultural perspectives and agendas as well as being decolonising,^33,35^ and this process requires significant changes in the way research projects are established, designed, and conducted, and their findings disseminated. In our project this response was explicitly undertaken through the composition of the research team, the selection of services, and the research methods used.

### Research Team

The research team comprised Aboriginal and non-Indigenous researchers (no staff identified as Torres Strait Islander), most of whom worked together previously and established trusted working relationships. The inclusion of non-Indigenous researchers has required constant conscious reflection on the role of Aboriginal and non-Indigenous researchers. We sought to balance the need for Indigenous sovereignty and control over research with the belief that non-Indigenous researchers need to contribute to addressing colonial systems. The non-Indigenous researchers sought to engage in the research in the spirit of Muller's call that “Decolonisation presents an invitation for the settler society to understand and acknowledge the process of colonisation and to collaborate in the decolonisation process with Indigenous peoples”.^40^^, p. 53^ We aimed to privilege Aboriginal team members’ voices and experiences, and to frequently revisit and reflect on the conduct of the project through team meetings.

### Selection of Services

Australia has Aboriginal community-controlled health services that are primarily funded by the Australian government (through multiple contract agreements and fee-for-service payments) but managed by elected community boards, and Aboriginal health services managed and funded by the government within state and territory government health department structures. We wanted to ensure both types of services were included in the research to understand what and how decolonising practices could be achieved in these different contexts. We were aware that including both community-controlled and state-managed services risked negative comparisons between the two sectors, as they may have considerable differences in their scope for action. The government-managed services are particularly aware of this risk, given they work within potentially colonizing government environments. It is important that they be able to trust that the research would not have a negative effect on them.

In our selection of services, we sought to continue our relationship with the two Aboriginal PHC organizations from the previous PHC project, and we sought a further three organizations to reflect the heterogeneity of the models of Aboriginal and Torres Strait Islander PHC in Australia. This representation was achieved through a mixture of professional networks and cold-call approaches. We sought community-controlled and government-managed services, from rural and metropolitan regions in different states and territories.

## Research Design

The research needed to be able to deal with the complexity of PHC services tailoring their ways of working to meet community need and context. The research also aimed to center and privilege Aboriginal and Torres Strait Islander voices and research methods.^41^ While initially framed as a mixed-methods research design, the project pivoted to privilege qualitative inquiry and yarning approaches^42^ to be responsive to requests from community-based partners to generate contextual knowledge on the nuances of decolonising practices. Methods were designed to draw on the experiences and knowledge of community and staff, both in groups (e.g., workshops and focus groups), and as individuals (e.g., staff interviews). Participants in all methods provided written informed consent.

### Cooperative Inquiry

We anchored the research in a cooperative inquiry model^43^ as a framework where partner health services could contribute as coresearchers, which aligns with decolonising methods and community-based participatory research. Cooperative inquiry is a form of participatory action research that emphasizes participants as researchers and intersects with Indigenous approaches to partnership-based research. Here, partner service staff were included as chief or associate investigators on the grant application, had input into the research design both before and after the grant submission, and contributed to interpretation and report of findings. Cooperative inquiry allows a “critical community of inquiry nested within a community of practice”^44^^, p. 172^ and has been used successfully in past Indigenous health research.^45,46^ We selected cooperative inquiry because of its capacity to recognize the importance of Aboriginal and Torres Strait Islander knowledge systems, cultural values, and lived experiences, centering these aspects as integral to the research process.

**Figure 1.**
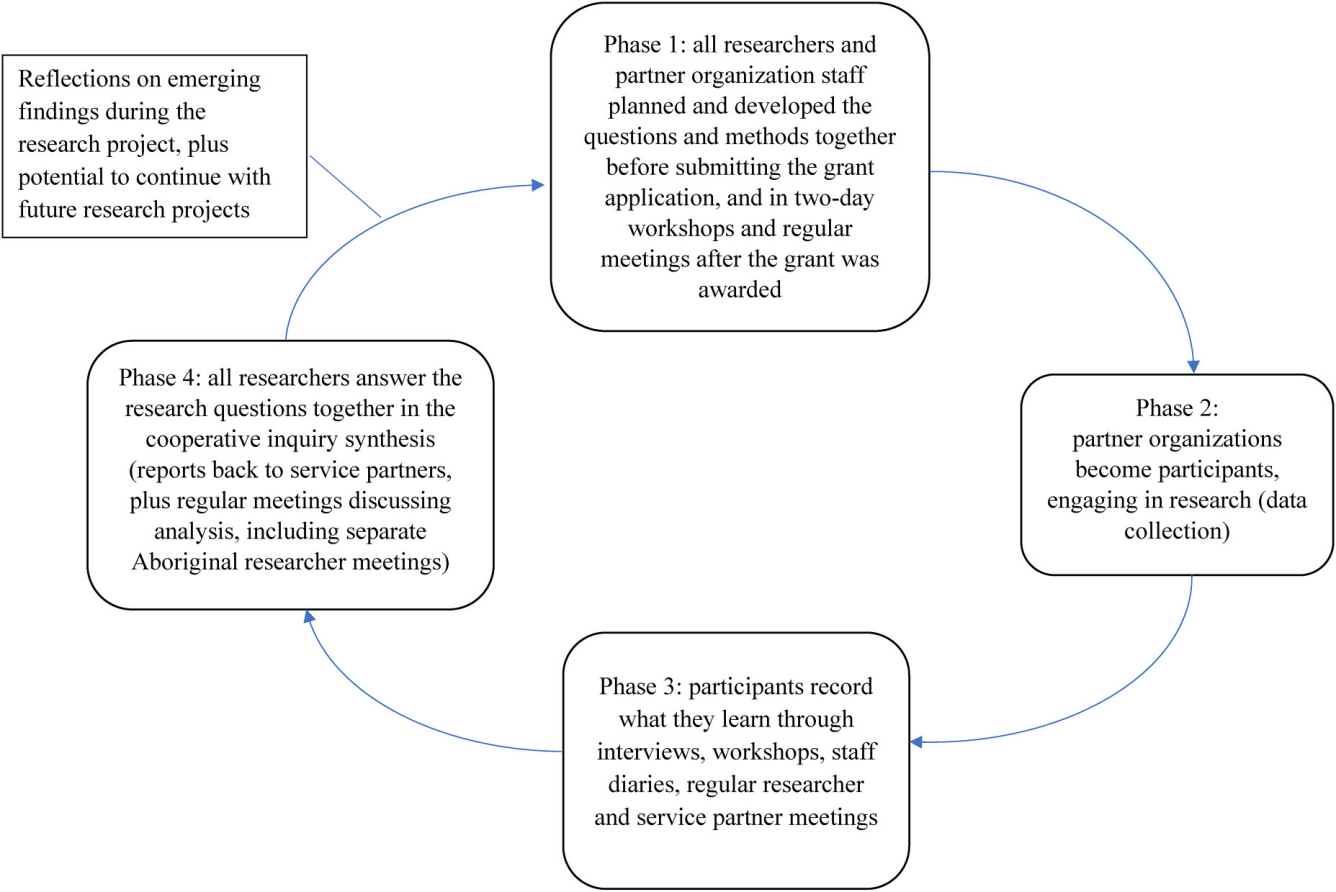
Cooperative inquiry method.

Cooperative inquiry involves four phases.^43^ In Phase 1, all researchers, including partner organization staff, planned and developed the questions and methods together. In this case, Phase 1 incorporated collaborating on the grant application and early research team meetings with all research partners to progress the detailed design and planning, including a two-day, face-to-face workshop with representatives from each of the partner services. In Phase 2, partner organizations became participants, engaging in research. To do this, we designed a range of data collection methods with the partner services and the communities they serve to address the research questions. In Phase 3, participants recorded their experiential knowledge arising from the research, and we captured this information through workshops and interviews with staff, staff diaries, and reflection and feedback at project meetings. In Phase 4, all researchers, including service partners, are contributing to answering the research questions together in the cooperative inquiry synthesis, drawing on propositional knowledge. How we implemented the cooperative inquiry method in our research project is summarized in Figure 1.

## Governance and Partnerships

We aimed to establish research methods and governance that reflected the objective of the research to partner collaboratively with health services so they could contribute to the research questions, design, interpretation, and reporting, and to ensure the research was culturally appropriate and relevant to their local community.

Regular researcher and partner meetings have been held each year to discuss research design, priority setting, data collection planning, analysis processes, emerging findings, academic publishing, and opportunities for impact and knowledge translation—the entire research process. These meetings have supported the research team's ability to maintain relevance to the field, to center service perspectives, and to make research decisions that are open and transparent to foster trust in the research process. A key outcome of this process was also a decision to focus the research on qualitative methods and yarning approaches,^42^ reflecting the team's commitment to be maximally responsive to input from community partners on how to generate new knowledge on decolonising practice, and health service partner advice about the most appropriate way to capture client views on decolonising practice.

Cooperative inquiry has also allowed collaboration and sharing of information learned between services. One example of the benefits of this method is that senior executives from one of the community-controlled health services who had extensive experience in decolonising practice were able to visit (prior to COVID-19 travel restrictions) and contribute to a decolonising practice workshop at one of the state-managed services. This collaboration has allowed a strong, reciprocal network to begin to grow around the research project.

In addition, we established a project advisory group comprising representatives of Aboriginal and Torres Strait Islander main bodies involved in the PHC sector and key health system actors who meet regularly over the course of the project and have assisted with research aims such as refining definitions of decolonising practice. Such advisory groups are valuable to health research because of their policy expertise and deep understanding of the field, connections with policy actor networks, advice to ensure the research remains relevant to policy and practice, and assistance with knowledge translation.^47^ We identified desired potential members from a mapping of the sector in Australia. Membership of the group has changed over time in response to personnel changes in the main bodies, and to include other relevant organizations. At each research team, service partner, and advisory group meeting, attendees can provide feedback on the governance of the research and ways of working.

## Part Two: Planning, Not the Plan

As the ethics processes for the project neared completion, the global pandemic of COVID-19 reached Australia. International and interstate borders closed, health services had to rapidly adapt their ways of working, and the high levels of uncertainty compromised all ability to plan. There was also a series of severe weather events in Australia during the research period that affected one or more of the partner services, impacting service priorities and data collection. A timeline of the research, and the COVID-19, and other events that affected the project are shown in Table 1.

**Table 1.** Timeline of Research Progress, Severe Weather Events, and COVID-19 in Australia.^48,49^

Date	Event
March 2017	Research grant submitted
November 2017	Notified of grant award
July 2018	Initial in-person meeting of all partner services and investigators
October 2019	Data collection commences after extensive ethics applications
December 2019	COVID-19 first reported in Wuhan, China
December 2019 – January 2020	Major bushfires in New South Wales South Coast (almost 500,000 hectares, 312 homes destroyed)
January 2020	First COVID-19 case reported in Australia
February 2020	Severe floods in New South Wales and Queensland
March 2020	Human biosecurity emergency declared in Australia; international and domestic border closures, social distancing restrictions, health service access restrictions enacted
February 2021	Vaccine rollout begins in Australia
March 2021	Floods in New South Wales
July 2021	National plan to transition to a post-vaccine COVID-19 response released
November 2021	University restructure sees disestablishment of lead research institute
February – March 2022	Severe Floods in Queensland and New South Wales
March 2022	Disestablished team members move to University of Adelaide

Despite these obstacles, we have managed to collect rich and valuable data through careful and trusting negotiation with partner services and a flexible approach to the research design, including data on how the services responded to the COVID-19 pandemic and how their decolonising practices helped safeguard the health of the Aboriginal and Torres Strait Islander communities they served during the pandemic and severe weather events. Data collection was also affected by health service, university, and staffing considerations.

### Health Service Considerations

As has been documented in the literature on health research during COVID-19,^20–23^ our health services research data collection needed to be immediately paused, and planned visits were cancelled. As well as the prohibitions on interstate travel, health services curtailed visitor access and suspended involvement in research while they focused on the emergency of COVID-19, all of which restricted any ability to undertake data collection.

As these initial border and health sector restrictions and health service activity stabilized somewhat into a more predictable state of affairs (though outbreaks, new COVID-19 variants and waves, lockdowns, and changing public health measures still created ongoing instability), what data collection was still possible during the pandemic became a difficult question. Spagnolo, Gautier, Seppey and D'Souza^21^ recommend incorporating participant perspectives into the decision making around adapting research methods in response to the pandemic. We found having established a flexible, cooperative inquiry design was a great benefit to discussion and negotiation on these issues in partnership with the health services. We held videoconference meetings with health service representatives to plan and go through data collection methods for each site to gauge their feasibility, and how they could be adapted. In relation to the whole project, we were able to have partner health service staff contribute to discussions as to what aspects of the research to prioritize, and which aspects would need to be relinquished or altered.

There have been constraints on flexibility of the research design and conduct, with the most pressing being the set amount of funding that was awarded, which flows on to the set amount of salaried staff time on the project. This constraint has meant that when requests have been made to alter elements of the research method, such as moving community forums off site from the partner health services to encourage more open conversation, funding parameters had to be balanced. However, we found the open research planning discussions fostered by our governance approach and cooperative inquiry have allowed these decisions and trade-offs to be made with clarity and with broad input.

We sought to not merely answer the question of how to adapt the research to allow it to continue. As Spagnolo, Gautier, Seppey and D'Souza^21^ frame the ethical dilemma, “Will the longer-term benefits of [the research] outweigh the short-term consequences (additional distress, working longer hours) on healthcare professionals and health planners as participants in the development and implementation of this type of research during a pandemic?” Health workers were widely documented during the pandemic to have high levels of distress, post-traumatic stress, and burnout.^50,51^ Cooperative inquiry allowed us to respond to this question as a team of researchers and service partners, choosing when and to what extent to halt research, and when to adapt it and continue it. Research only continued because health services recognized and appreciated its value, held a strong sense of control over its implementation at their respective study sites, and firmly believed that the data was worth pursuing despite the prevailing circumstances. Their commitment and endorsement of the research played a crucial role in ensuring its continuity and is a testament to the flexibility of the people working in the Aboriginal and Torres Strait Islander health service sector, who have long been innovative pioneers in PHC governance, design, and implementation.^9^

Notable examples of research adaptation included merging similar data collection methods together, particularly for engaging community members. We converted several staff workshops to online using videoconferencing. To maximize data collection within limited resources, we compressed timelines and consolidated multiple site visits into single, intensive visits, which originally spanned multiple trips. These adaptations were implemented to streamline processes and maximize the research outcomes within the constraints. Some elements of the research plan, particularly an ambitious multi-wave survey of service users, had to be cancelled as they were deemed no longer feasible. The cancelations were a response to COVID-19 restrictions and patient safety and to reduce the research burden on overstressed health services. We sought to remain true to decolonising research methods over this time, prioritizing the considerations of the communities, the services, and the Aboriginal team members in the decision-making process.

### University Considerations

The effects of the pandemic and severe weather events were felt by universities as well as health services. During COVID-19, universities coped with restrictions by reorienting teaching to online, and encouraging staff to work from home. Prior to COVID-19 universities were reported by many staff to be difficult workplaces, with low psychosocial safety, and this was likely exacerbated during the early COVID-19 period.^52^ International students were unable to travel to Australia to study, triggering a perceived funding crisis for Australian universities where international students form a significant proportion of universities’ revenue.^53^ This perceived funding shortfall was used as a justification for widespread job losses and restructuring across the sector, despite universities continuing to record substantial surpluses.^53^ During the pandemic, the main institute leading this research was “disestablished” by its host university, with researchers including a tenured staff member losing their jobs. While these researchers were able to re-establish their work at another university, others remained at the original university thereby splitting the core team. The considerable time spent on the transition, learning new systems, and transferring research funding and ethics approval to the new university all impacted the productivity of the research.

### Staffing Considerations

The project funder, the Australian National Health and Medical Research Council, announced 12-month extensions to all research projects due to disruptions associated with the COVID-19 pandemic, but the extension did not come with extra salary funding. No extra staff time was available for the research. We found while less staff time was spent directly collecting data, more time was spent on negotiations with services and research planning. On top of this, consistent with previous reporting in the literature,^20–24^ there was a considerable toll on staff from the pandemic and lockdowns. Staff members contracted COVID-19, and some Aboriginal staff had COVID-19 outbreaks in their communities. Staff with young children found themselves working from home and caring for children, and/or home-schooling during lockdowns, while other staff also faced caring responsibilities for family members. University work including teaching, meetings, and committees were required to move online, which entailed additional organization and planning. Researchers reported that stress, uncertainty, management pressure, and competing demands were very high during this time. The research team member situated in one of the health services was redeployed to respond to COVID-19 needs. Planned interstate site visits, when they resumed, sometimes had to be cancelled because of illness or sudden border closures. All these factors reduced the amount of research that could be completed.

Additional burdens and the fraught nature of navigating overwork and racism for Aboriginal and Torres Strait Islander academics have been documented,^54^ and our experience indicates that the disruptions COVID-19 brought to universities continued and exacerbated this power dynamic. The burdens on Aboriginal and Torres Strait Islander academics often intersect with the complexities of their cultural identities and responsibilities within their communities. The ongoing power dynamics stemming from these disruptions highlight the need for universities to address systemic inequities and provide targeted support to ensure the well-being and success of Aboriginal and Torres Strait Islander academics. Recognizing and addressing power differentials is essential to ensure equitable participation, meaningful engagement, and meaningful research outcomes.

Despite these extra time costs, the data collection pause that the COVID-19 restrictions necessitated also made time available to the research team that we may not otherwise have had. As others found,^21,23^ we were able to use this time to allow deeper reflection than may have otherwise been possible, revising methods and talking through ideas. In our case, this time allowed more considered, in-depth Aboriginal-led analysis of the data we had already collected than may have been possible under the original data collection regimen, allowing us to better pursue decolonising research methods. Thus, while the amount of data collected may have been reduced, we have found the quality of the analysis to have increased.

### Challenges of Cooperative Inquiry

We found, and continue to find, cooperative inquiry to be a highly valuable, partnership-focused approach to health services research. However, it did present some challenges.

#### Time Investment

Cooperative inquiry has necessitated discussing decisions with the broad group of academics and service partners, which can be time intensive. The time burden of participation is perhaps greater for service partners than less cooperative research, but with the intended trade-off that the research conducted is more fit for purpose and relevant to the organization's goals. When turnover occurred in the service or research team, the briefing of new personnel and supporting them to contribute to the project may have been more time intensive than for other projects. For the academics, cooperative inquiry meant a large time investment in relationship building and democratic discussion, which is work often rendered invisible in university metrics that typically value concrete outputs such as journal articles. These institutional dynamics and values have previously been highlighted as reproducing colonial power relationships and research approaches.^55^

#### Ethical Approvals

Related to time investment, ethical approval may provide further challenges to cooperative inquiry research. While ethics applications and associated research governance processes are known to take a large amount of staff time to complete for all health services research, the collaborative nature of the research may have made it more difficult to fit the research into ethics committee forms and requirements, and in some cases, multiple revisions were needed before the ethics committee would approve the project. One ethical dilemma was that of dual roles. One of the researchers on the project is employed at one of the partner health services, and one of the Chief Investigators also had a position at the same health service. This has worked well to keep the health service informed of and engaged in the research. It has also been valuable in allowing us to tailor the design and data collection using local knowledge and contributes to building capacity for research at the health service. However, it has also led to difficulties in thinking through the ethical issue of what data the researcher and investigator ought or ought not to have access to in order to contribute to collaborative decision-making in the context of cooperative inquiry, and when it is appropriate or inappropriate for them to conduct data collection at the health service.

Further, as methods were debated and altered through cooperative inquiry, each change in methods required ethics modifications to the multiple ethics committees involved. The time taken for these processes was a significant cost to the project.

#### Mismatch Between Cooperative Inquiry and Research Funding Processes

Cooperative inquiry and other participatory or partnership-based methods often rightly emphasize the need to work with partners in developing a research agenda and designing research projects prior to writing a grant application.^44^ Consequently, and in the context of long funding cycles and low grant success rates, there was a long lag time between the first approach to the services and winning research funding to initiate the project. The lag time made it difficult to maintain relationships with the services. Over time, two services withdrew—one prior to and the other after funding was awarded due to personnel changes and insufficient capacity to participate. This situation reflects the broader context of service priorities, timelines, and competing pressures. Two new services were recruited to join the research project. In addition, cooperative inquiry means that methodological processes need to be flexible to respond to ongoing input. However, grant bodies generally require the methodological approach to be outlined in full, leading to a tension between the certainty required by granting bodies and the need for flexibility in cooperative inquiry.

Despite these challenges, we found considerable benefits from cooperative inquiry when navigating the uncertain times of the COVID-19 pandemic and series of severe weather events. We continued to collect data throughout this period in negotiation with the services, and have begun collaboratively generating new knowledge with research findings that are relevant to policy and practice, and that honor the decolonising aims of the research and reflect the experiences and priorities of the health service partners.

## Conclusion

Health services research is fundamentally a partnership between researchers and health services, even when those roles overlap, characterized by mutual engagement, shared goals and a commitment to improving health. We have very little data on what has happened to health services research during the COVID-19 pandemic. While we have faced difficulties in this research project, our experience highlights that participatory multi-state projects based on partnerships with health services are possible. We argue that the potential benefits of such partnership-based research are responsiveness, the full incorporation of the experiential knowledge of the health services in all aspects of the research process, and the development and translation of knowledge of what works. Achieveing these benefits will require increased use of cooperative inquiry and other participatory methods in health service research, supported by inclusive governance structures overseeing the research, and deep reflection from all research contributors. This approach has not always been the traditional path to health services research, and there are drivers in both the academic sector and health sector that could be altered to support such research. Our experience suggests this approach is valuable with great benefits for health systems, universities, funders, and ultimately the community. It also makes a crucial contribution to decolonising Indigenous health research.

The COVID-19 pandemic is not a temporary blip on the landscape of health services research, but rather is likely to signal greater need in the future for flexible research that responds to concurrent crises. The frequency of severe weather events, the health impacts of climate change, and new emerging zoonotic diseases will only increase.^56,57^ For all of these reasons, flexible, participatory methods such as cooperative inquiry will continue to be vital for successful health services research predicated on partnerships between researchers and health services into the future.
